# Multidrug-Resistant TB in the Philippines: Authors' Reply

**DOI:** 10.1371/journal.pmed.0030549

**Published:** 2006-12-26

**Authors:** Thelma Tupasi

The comments of Garner et al. [1] and Portero and Rubio [2] on our article, “Feasibility and Cost-Effectiveness of Treating Multidrug-Resistant Tuberculosis: A Cohort Study in the Philippines” [3] warrant a response. This reply is written on behalf of all authors of the original article.

Garner et al. focus on four issues: 1) lack of independence because of author affiliations and financial support from the Global Fund to fight AIDS, TB and Malaria (GFATM); 2) whether results can be replicated beyond the pilot project that was studied; 3) the need to pay attention to health system capacity; and 4) financial sustainability. Portero and Rubio state that Makati Medical Center (MMC) is a tertiary institution operating under “ideal” conditions not typical of other health facilities, and that lack of attention to building laboratory capacity may hinder service expansion. They also argue that a community-based care approach should have been studied at the pilot stage.

The standard methods used for statistical analysis and economic evaluation in our study [4–6], a rigorous peer-review process, and the application of previously established methods for the evaluation of multidrug-resistant tuberculosis (MDR-TB) treatment [7] provide for objectivity in the results that are reported. We strongly disagree with the insinuation that the participation of the World Health Organization or funding from the GFATM biased either the analysis or the way in which results were reported.

Pilot projects are often an important first step in implementing and evaluating new interventions. At the same time, it is well recognized that results can be different when services are scaled up. We agree that care is required when interpreting results from pilot projects, and that evaluation of MDR-TB treatment when implemented at a larger scale is necessary. Our article identifies the factors that we believe need to be replicated if results are to be reproduced elsewhere. In the meantime, it is important to highlight that while housed in a tertiary facility, the project was based in an outpatient clinic; that the cost-effectiveness results are consistent with those for a national programme in Peru [7]; and that community-based care is now available. Since 2004, patients have been treated at their local public health centre, which has helped to increase cure rates and reduce default rates compared to those observed during the study period [8].

We agree that health system capacity should be considered when new interventions are implemented. In the Philippines, expansion of MDR-TB treatment has been planned such that treatment sites will typically manage one to three patients at any given time. Decentralization of services to a larger number of treatment sites is helping to integrate MDR-TB treatment into existing TB treatment services provided at primary health-care level, while increasing accessibility for patients. Treatment sites will be overseen by larger treatment centres, selected according to various criteria including their capacity and willingness to take on additional responsibilities. Laboratory and human resource capacity are being enhanced at treatment centres and treatment sites, just as capacity was enhanced at MMC during the pilot phase. Such costs (including infrastructure and training) were included in our cost-effectiveness analysis, and Garner et al. are incorrect when they state that they were not. All resources used in the start-up and implementation phases of the project were costed, in line with standard guidelines [4].

It is true that most of the current funding for MDR-TB treatment is from the GFATM. However, the government has provided additional funds for TB control, including MDR-TB treatment, since 2005. Funding for the treatment of new drug-susceptible cases has not been reduced. This demonstrates that MDR-TB treatment can be provided without compromising basic tuberculosis control. Indeed, some of the investments needed for expansion of MDR-TB treatment, such as building laboratory capacity, may strengthen the health system as a whole.

Regarding more specific criticisms [2], the lack of HIV testing and long-term follow-up are acknowledged in our article (though HIV prevalence remains low, at less than 0.1%) [9]. We specifically compared patients who were enrolled with patients who were eligible but not enrolled. The two groups were similar, except that enrolled cases had more, rather than less, severe patterns of drug resistance. Results are referred to in our article and reported in supplementary material. As reported in our article, we analyzed the relationship between several variables and treatment outcomes; the only statistically significant result was that women appeared more likely to be cured than men. Also, contrary to the claim made by Portero and Rubio, Resch et al. made no suggestion that our study lacked independence [10].

In conclusion, the overall goal of our study was to contribute to the evidence base on the management of MDR-TB in low- and middle-income countries. We support the development and implementation of evidence-based management strategies for all TB patients.
